# Fourteen quick tips for crowdsourcing geographically linked data for public health advocacy

**DOI:** 10.1371/journal.pcbi.1011285

**Published:** 2023-09-21

**Authors:** Joshua Atienza, Anjalee Benedict, Lincoln D. Stein, Kashif Pirzada, Cheryl White, Shraddha Pai

**Affiliations:** 1 London School of Economics (School of Public Policy), University of Toronto (Munk School), Toronto, Canada; 2 University of Toronto (St George Campus), Toronto, Canada; 3 Ontario Institute for Cancer Research, Toronto, Canada; 4 Department of Molecular Genetics, University of Toronto, Canada; 5 Faculty of Health Sciences, McMaster University, Hamilton, Canada; 6 Community Access to Ventilation Information (CAVI), Toronto, Canada; 7 Department of Molecular Biophysics, University of Toronto, Toronto, Canada; SIB Swiss Institute of Bioinformatics, SWITZERLAND

## Abstract

This article presents 14 quick tips to build a team to crowdsource data for public health advocacy. It includes tips around team building and logistics, infrastructure setup, media and industry outreach, and project wrap-up and archival for posterity.

## Introduction

The need to collect data linked to geographic location can arise in many disciplines including infectious disease epidemiology, biology, health, social science research, and public health advocacy. Crowdsourced data on a microblogging social media platform such as Twitter has a recognized role in directing dynamic individual and community emergency response during natural disasters [1–3], and in capturing COVID-19 infection dynamics in community settings for public health advocacy [4,5]. Citizen-led projects that organically arise during times of crises situations must combine an organizational structure with the technological infrastructure and agility to affect change.

**Fig 1:**
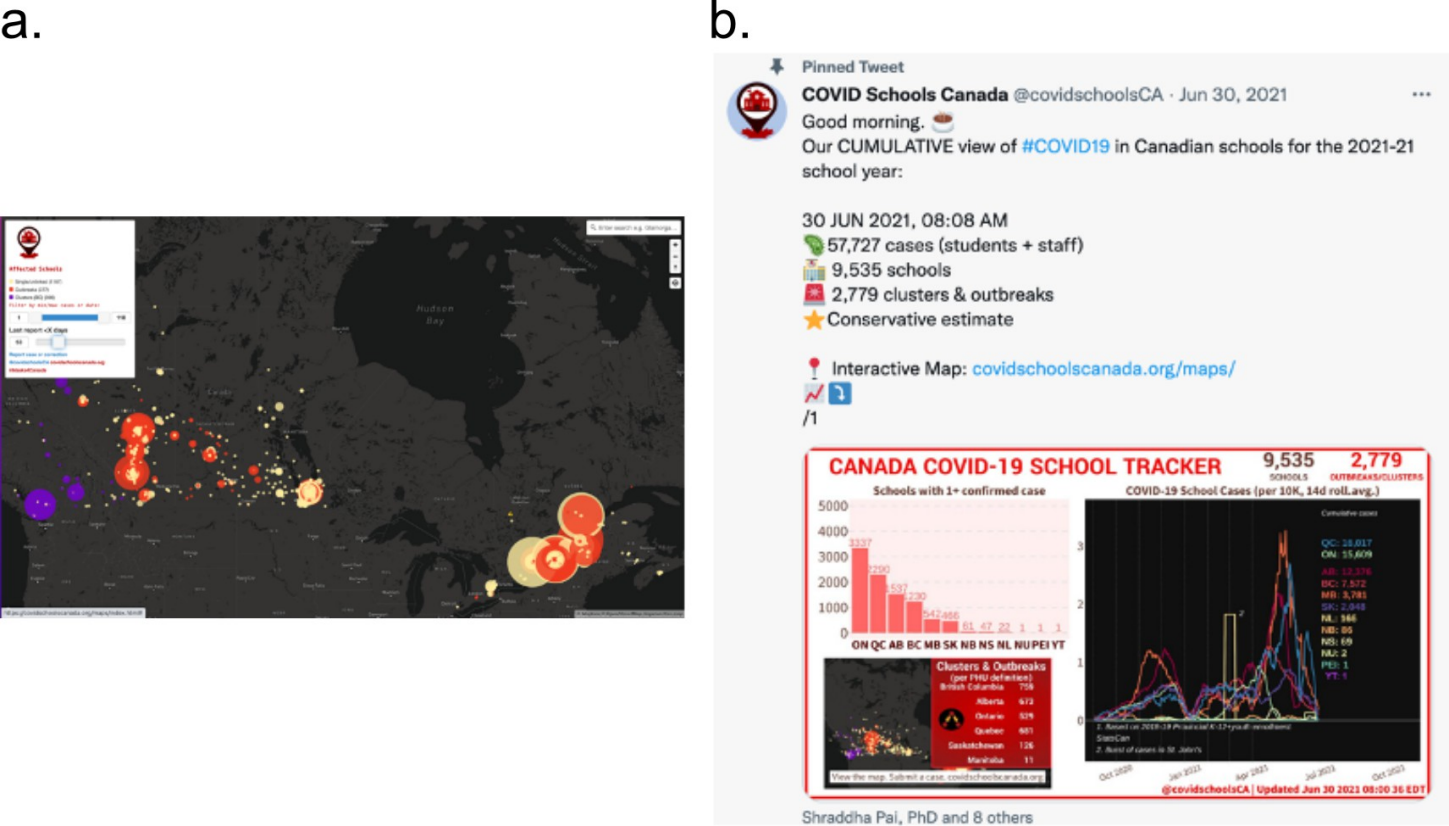
(**a**) View of interactive map displaying COVID-19 cases and outbreaks across schools in Canada, collected by COVID Schools Canada (covidschoolscanada.org). Map generated using MapBox (https://www.mapbox.com/). (**b**) Example view of code-generated tweet displaying summary statistics.

This article provides a guide to creating a crowdsourced data gathering project and advocacy. We will cover team-building logistics, technology, and infrastructure for data storage, visualization and dissemination, and recommendations for public and media outreach (Table 1). These tips are based on our experience creating and running the grassroots initiative Covid Schools Canada (CSC; covidschoolscanada.org). CSC arose organically on Twitter to advocate for public health risk mitigation and crowdsourced nearly 58,000 COVID-19 cases and 2,800 outbreaks in Canadian schools from September 2020 to June 2021 (Fig 1) [5]. The project provided daily data updates to 10K+ Twitter followers, gave nearly 40 broadcast and print interviews in Canadian and US news outlets [6–12], and CSC data have been used to model the impact of specific interventions in Canadian schools [4]. We hope that this guide will empower similar community projects in service of evidence-based public health advocacy, and we have provided links to the CSC project code and data on an as-is basis at the end of this article.

**Table 1:** Quick tips to crowdsource data for public health advocacy.

**Team and Collaborator Logistics** 1. Assemble a team using existing online and offline community hubs. 2. Establish team roles, and communication protocols and channels. 3. Collaborate with regional experts and data gatherers.	**Technology and Infrastructure** 4. Design a software pipeline for timely data updates and visualization. 5. Create shared assets and a web presence for the project. 6. Create a strategy for data gathering using “divide and conquer”. 7. Build a cloud-based directory structure amenable to automated data harvesting, and define data entry protocols. 8. Use web scraping libraries to automate data harvesting. 9. Create a clearly advertised channel for grassroots crowdsourcing. 10. Use geocoding libraries and GeoJSON to put data points on a geographical map.	**Outreach** 11. Find the right social media platform. 12. Give broadcast and print media interviews. 13. Build partnerships with industry to improve your platform. 14. Know when to end the project and be open to future collaborations.

## Team and collaborator logistics

### Tip 1: Assemble a team using existing online and offline community hubs

Project leads should consider the skills, motivations, and diversity of experience required to form a cohesive team. Different educational backgrounds, work experiences, and language proficiencies can offer flexibility and fresh perspectives to drive your project. Leveraging existing community groups will help grow a team in relatively short time and avoid the energy spent in starting a new endeavor that never gains critical mass. Professional networks on social media platforms can facilitate team recruitment. Advocacy groups can also partner with academic mentorship and skill building programs—such as those in university settings—to recruit students. Students will gain learning opportunities while contributing to a project with population health benefits. In this view, the team will benefit from a combination of mentors and learners to execute the project. Advocacy groups must establish protocols agreed upon by team members for recruiting new members as needed or on a rolling basis.

CSC benefited from starting as a subgroup of the Masks4Canada initiative and by being, to our knowledge, the first to seek to crowdsource school-related data across the country for advocacy. It leveraged Masks4Canada, a network of diverse experts—physicians, lawyers, engineers, scientists, and other concerned citizens—who found each other on Twitter in early 2020 as regular Twitter users with a shared passion for promoting evidence-based public health policy in Canada during the COVID-19 pandemic. Founding members had previous expertise in crowdsourcing and crowdfunding for public health, and media outreach for advocacy, and gained attention via a cohesive message, media outreach, open letters, educational posts on social media, and consistent branding. This team built a volunteer base of undergraduate students from the Community of Support program run through University of Toronto Medicine and still continues to recruit technical experts through social media interactions.

### Tip 2: Establish team roles, and communication protocols and channels

Initial collaborator meetings are important for setting the tone and context for your collaboration. It can clarify the scope of the project, provide training on data management practices, and enforce team conduct expectations. While project founders are usually initial leaders, establish co-leads based on domain expertise. Establish a protocol for meetings and consensus building (e.g., using Martha’s Rules) [13]. For team communication, set up a chat server on one or more messaging platforms (e.g., Slack, Discord, WhatsApp, Signal). Some experimentation may be needed to find which platform works best for the team. Agree on a schedule for a recurring meeting with a formalized agenda, stored in a shared document space (see Tip 7), and record minutes. Meetings foster regular project follow-up and commitment, collaboration among team members for troubleshooting, and create camaraderie. Employing focused team-oriented approaches to goal setting and task allocation, such as those espoused by the Agile framework [14], and using tools such as Kanban boards, may ensure equitable work distribution among team members and prevent bottlenecks. At CSC, Newcomers were either technical experts who joined the group with a specific contribution in mind, or were undergraduate trainees looking to volunteer in any capacity. Onboarding of trainees was achieved by identifying which sub-team a person would work on, what their contribution would be, and by making their first jobs low risk, small in scope and/or repetitive. They were paired with a more experienced team member who would instruct them in a one-on-one session, and their need to learn was limited to their immediate contribution. Where there was a manual (e.g., how to enter new cases), they were asked to read subsections relevant to their contribution. Once a new team member mastered one task, they would take on larger responsibilities and/or train others. Ideas from onboarding in the manufacturing sector may be useful here (Training Within Industry [15]).

### Tip 3: Collaborate with regional experts and data gatherers

Consistent social media presence (see Tip 11) can raise awareness about your advocacy initiatives and establish buy-in from other data collaborators and experts to build traction for your project. Data can be crowdsourced directly at the individual level, and indirectly from regional crowdsourcing initiatives. For individual contributions, create a method that makes it easy and secure to submit data, such as a website form, an app, a map, an email address, or a phone number to call or text (see Tip 9). For data collected by regional experts or organizations, make sure at the outset that their data are compatible and can be implemented into your infrastructure. Consider if these sources are recording the same data variables and at the same granularity, and whether there is duplication between the two groups. Reach out to project leads to initiate a collaboration, clarify the nature of the data, and request permission to set up routine data mirroring. CSC collaborated with province-level crowdsourcing initiatives in Québec (COVID Écoles Québec), Alberta (Support Our Students Alberta), Saskatchewan (Safe Schools Saskatchewan), and British Columbia (BC School COVID Tracker).

## Technology and infrastructure

### Tip 4: Design a software pipeline for timely data updates and visualization

**Fig 2:**
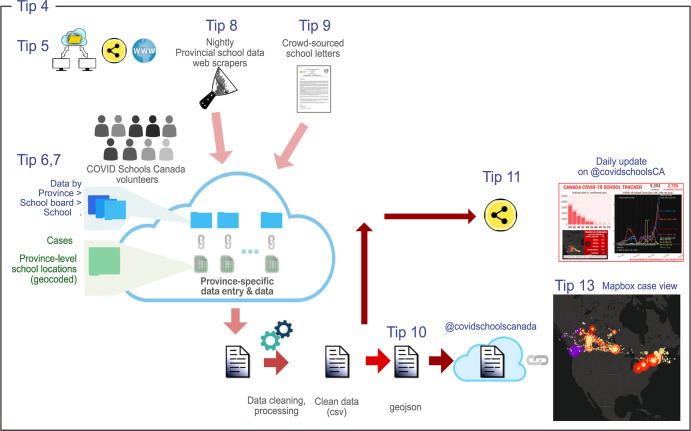
Workflow used by COVID Schools Canada (CSC) to crowdsource COVID-19 cases and outbreaks data across Canadian schools. Data were collected using a combination of automated web scrapers (Tip 8), manual spreadsheet entry from a combination of school board and province-level websites (Tips 6 and 7), and from a Google form on the CSC website for individual reports (Tip 9). Custom R code was used to pull data from these varied sources, clean the data, and assign latitude/longitude coordinates to each school entry (Tip 10). The final CSV file was converted to GeoJSON format and pushed to GitHub. Mapbox was used to create the interactive map and automatically refreshed its view from the file on GitHub (Tip 13). Table 2 lists all major software packages used for building the pipeline. Map generated using MapBox (https://www.mapbox.com/).

A software pipeline can help routinely clean, analyze, and visualize collected data for public dissemination (e.g., social media). Fig 2 shows the CSC pipeline. This workflow is simplest if all entered data are hosted on a cloud-based system such as Google Drive (Tips 5 and 7). With your team, agree on a daily time by which manual entry for the night should be complete, coordinating on a chat channel for possible delays. Using your programming language of choice, create a script that uses application programming interfaces (APIs) that pull updated data from the cloud on to your computer; cleans the data, checking for missing values/data entry errors; identifies coordinates for each data point; aggregates statistics; creates visualizations for public dissemination; generates social media posts with key statistics; and creates a nightly data freeze, which gets pushed to the cloud. Complete pipeline automation may not be possible if data are manually entered, as not all error-handling scenarios can be anticipated a priori, or if data are scraped from diverse online sources with inconsistent or changing formatting. The CSC nightly build is publicly available (see Data availability). Table 2 lists major software packages used for this pipeline, and all URLs are included in S1 Text.

**Table 2:** Technologies used for Covid Schools Canada. We recommend teams reach for tools they already know, and where there is an intractable gap, recruit technical expertise to help overcome it. See S1 Text for associated URLs.

Category
**Communications and online presence** • Team Communications: Chat: WhatsApp, Slack, Signal; Videoconferences: Zoom • Social media: Twitter, TikTok, Facebook • Website creation (free): GitHub Pages, Jekyll Themes
**Software packages and online platforms for mapping data** • Create custom map: Google Maps (up to 10K data points) • Create Javascript-based interactive maps, free tier and technical support for volunteer groups: MapBox • Geocoding: *photon*, *mapboxapi* if using MapBox; *ggmap* if using Google Map API • Analyzing geospatial data: *geopandas* in Python
**Data harvesting and broadcasting:** • Data hosting: Google Drive, Dropbox, Amazon Web Services, linked to Gmail account for project • APIs to pull cloud-hosted data: *gsheets* for Google Sheets; *rdrop2* for Dropbox; *boto* for Amazon Web Services • Scraping static HTML packages: Perl packages *libhtml-tableextractor-perl*, *libwww-perl*, *liblwp-protocol-https-perl*; *selenium* in Python • Scraping dynamic, Javascript-generated pages or to simulate user interaction: *nodejs* with *puppeteer* • Alert system for news reports with keywords of interest: Google Alerts • Working with Excel files: xlsx or *readxl* in R; *xlrd* in Python • Data analysis and visualization: *dplyr*, *ggplot* in R; *pandas & numpy* in Python • Tweet generation: *emo* R package for emoji generation • Code management: GitHub • Data freeze: Zenodo

To ensure high data quality, the pipeline should contain a set of code assertions anticipating common data entry errors, as well as data visualizations to help catch unanticipated errors:

- Typographical errors can be limited by checking proper names against a precompiled master list (e.g., names of institutions).
- Errors in entering dates and numbers can be limited by using assertions that ensure recency, values within a certain range (e.g., errors for negative values), or a numerical change within an acceptable threshold (e.g., “100 cases added, please check”)
- Missing values from omission or bad conversion of a string to a number can trigger errors.
- Assertions can be used to check that every data column matches its intended format.
- Create a set of graphs that slice the data along key axes, and show tallies for various geographic regions. Manually inspect this graph on a daily basis to catch errors.

Errors that cannot be automatically resolved will need to be manually investigated, and team members can be coached to avoid making the same kind of data entry error. As the project progresses, respond to new patterns of erroneous data entry using a combination of automatic data cleaning where possible and improved protocols for data entry.

### Tip 5: Create shared assets and a web presence for the project

Your team will need dedicated space for shared documents, contributed software (such as those used to generate data graphs), a dedicated e-mail address for the project, and a project website. For CSC, we used Gsuite (now Google Workspace) because it provided a low-cost, highly popular infrastructure for collaborative project documents. Using tools most people are familiar with reduces training overhead and makes the project more approachable to new team members. Google Drive also allowed us to share publicly accessible URLs for verification documents, which we used to create external links to supporting documents from individual data points on the interactive map of case reports. Creating a Gmail account associated with the project facilitated access to other Google Drive applications such as Google Forms, Google Docs, and Google Sheets. In the interest of data protection, we assigned at least 2 superusers as the main account managers to oversee member access control, password access, and manage file sharing permissions. We followed the principle of providing access on an as-needed basis.

The project website will host the geographical map, present the team, and list milestones and media appearances. Services such as WordPress and Squarespace can help create a website in a matter of hours with no technical work, allowing information to be displayed to the public quickly and efficiently. Users comfortable with programming with HTML and CSS can use Jekyll or Eleventy to help build the website and host a static site at no cost using GitHub Pages or Netlify.

### Tip 6: Create a strategy for data gathering using “divide and conquer”

Define the scope and granularity of data gathering, and identify natural geographic and institutional subcategories, and their relative population size. For example, Canadian schools are governed at the level of Province; therefore, CSC used a 3-tier level of data organization at the level of Province, school board, and individual school. Government’s open data websites may provide a ready-to-use list of categorized institutions. We used this hierarchy of data gathering to create region-specific data gathering teams and create a file structure in the project data store.

The next step is to decide how to delegate team members to specific geographic regions. Automating data for high-volume regions via scheduled web-scraping (Tip 8) will limit the burden of manual data collection. The quantity and quality of precollected data may vary among geographic regions as a result of variations in data reporting policies and responsible authorities. For example, the CSC project was able to automate most data collection for Ontario, as the province mandated official reporting of cases and outbreaks on school board websites, and with other Province-level crowdsourcing initiatives (see Tip 3). However, other provinces such as Manitoba required manual data collection of individual cases as there was no community project that had created a database of crowdsourced cases. We recommend that, where possible, the team identify strategies to solve problems upstream of data collection, by building collaborations with maintainers of source databases (see Tip 3). Identifying what is automatable and what is not can help recruit volunteers with suitable technical skills for automation and divide the responsibility of manual data collection. Delegate regions to team members according to individual ability to commit time and energy, and anticipated load.

### Tip 7: Build a cloud-based directory structure to automate data harvesting and define data entry protocols

**Fig 3:**
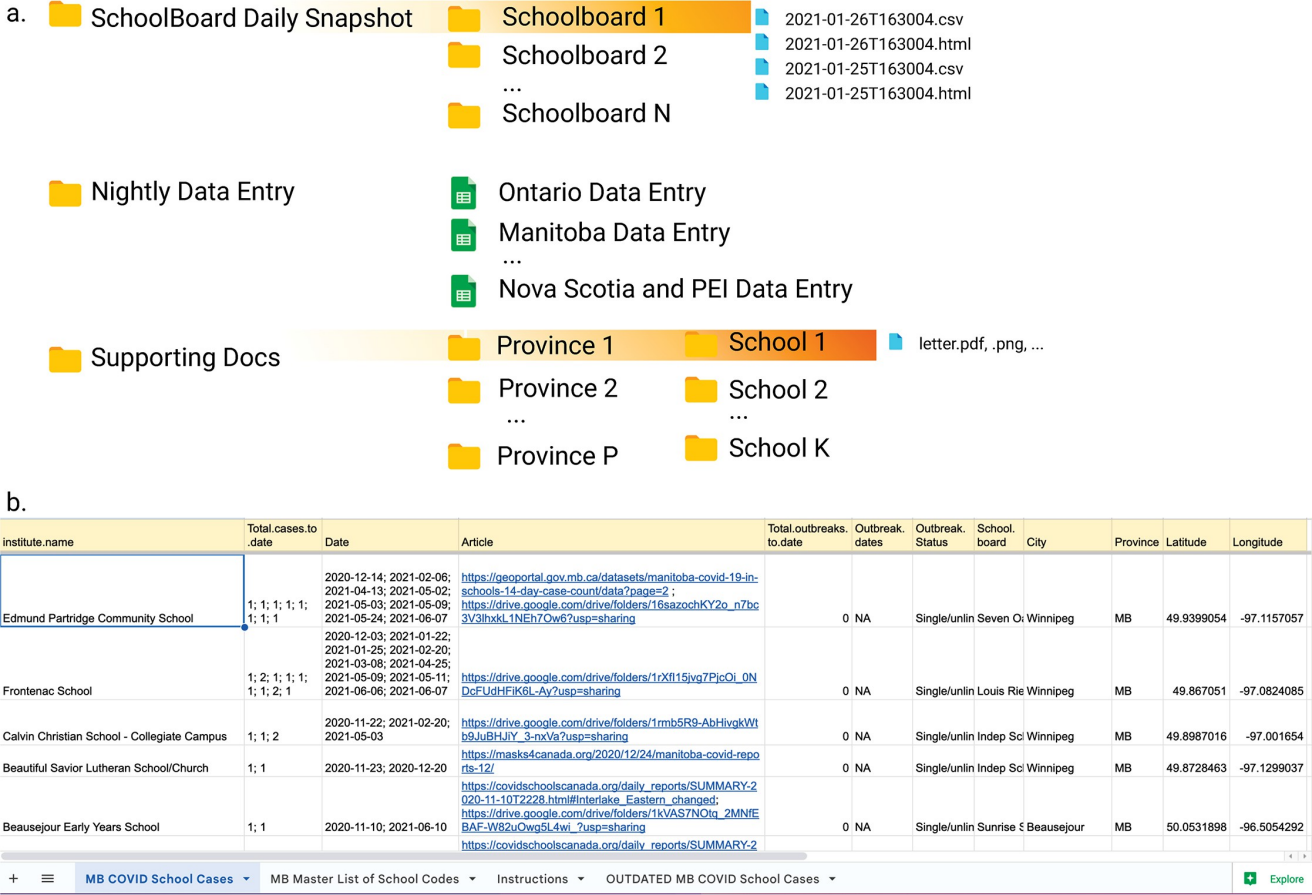
Example of centralized data storage for a crowdsourcing project and shared data entry table. (**a**) Directory tree on Google Drive containing web-scraped data (“Schoolboard Daily Snapshot”), spreadsheets for manual data entry (“Nightly Data Entry”; see detail in b), and supporting documents for case reports (“Supporting Docs”). Created using Biorender.com. (**b**) Example of a manual data entry file for the COVID Schools Canada project. Fields are standardized across spreadsheets, and the “Instructions” tab contains a reference for data for each field. The sheet contains a master list of school names, addresses, and geographic coordinates, which team members copy-pasted to create new case entries and minimize errors due to typing (“Master List of School Codes”).

The shared team project directory structure is a centralized location for members to enter data in master files, along with supporting documentation. To create an automated setup for data gathering, first create a master table of all entities at the level of highest granularity, along with their categorical levels (Fig 3A). This list will serve as controlled vocabulary for the project. Use this nested structure to autogenerate a directory structure for data deposition. For example, the first column will generate the first tier of folders, the second will generate the tier nested in the first, and so forth. If data directories are stored on Google Drive, one possibility is to create the master table using Google Spreadsheets and write an R script that uses the *googleAuthR* package to fetch the sheet and create a corresponding nested directory structure in Google Drive.

Create workflows and data entry protocols to pipe in data from automatically scraped and manually entered data. Scripts can save web-scraped data tables to the corresponding subfolder alongside verifying documents; e.g., data scraped from each school board lives in the corresponding folder name. For manual entry, create a master cloud-based spreadsheet for each major geographic division (e.g., per Province or State), with a uniform table schema containing all the fields needed for data collection (Fig 3B). Create a document with a data entry protocol, which indicates valid values for each field (e.g., date format), and walk through the document with the team; include validation triggers in the sheet if possible. Create a strategy to limit the need for volunteers to type in institution names, such as having a second sheet in the workbook with the master list. Team members can then simply copy-paste institution information and geolocations from the master list into the data entry sheet, thus reducing burden and error.

### Tip 8: Use web scraping libraries to automate data harvesting

We wrote a software framework to automatically harvest information on COVID-19 cases from Ontario-based public and private school systems, as the Province mandated data reports. Nearly all school boards we monitored had a website from which this information could be obtained. However, these data were reported in a wide variety of formats, ranging from easily scrapable HTML tables to challenging Javascript-based dynamically generated pages.

Our web-scraping software framework (see “Code and data availability”) was organized around a series of Perl modules specialized for different types of school district web pages. Using the Perl language’s inheritance mechanism, we wrote generic modules first, such as one for static HTML tables, and then wrote modules that handled school board-specific idiosyncrasies. We created a cron job to trigger a nightly scraper script that iterated through each of the board-specific modules, converted the data into a standardized tabular format (CSV), and then wrote the converted data, along with the raw HTML source, to disk. These standardized files were picked up by other scripts, which updated the Google spreadsheet described in Tip 7, and made a daily summary available at the project website. See Box 1 for a discussion of the pros and cons of using multiple programming languages within a group project.

**Box 1. Why did we use multiple programming languages for automation?**

Readers will notice that this project relied on several different programming languages for its automation, including R, Python, Perl, and JavaScript. This was an organic decision for a project that drew on the diverse background of multiple individuals to bring up a working system in a time-critical manner. The advantage of this approach was its speed and flexibility. Each programming language has its own particular strengths: for example, R’s prowess in statistical analysis, Python’s deep bench of data science libraries, and Perl’s easy integration with Unix command-line tools. By allowing our volunteers to choose the programming languages and libraries with which they were most familiar, we were able to rapidly launch a working system. The disadvantage of this choice is that the system as a whole became harder to understand and maintain, and one member of the team could not easily fill in for another when bug hunting or adding features. If the system we built were to be adopted for long-term use, it would be desirable to simplify the system to rely on one, or at most two, programming languages. Similar reasoning applies to other aspects of the project, such as our use of multiple cloud storage services to curate and exchange data.

There were 3 main challenges that we encountered with this part of the system. The first challenge was that school boards frequently changed the format of their case report pages, necessitating minor to major code changes. To catch these, we set up an error reporting system in which a daily e-mail was sent to the developer of the scraping scripts, summarizing the success or failure status of each school board. The second challenge was that there are many French language schools in Ontario, and we found that the handling of accented characters was wildly inconsistent from one school’s website to another. This necessitated a series of special cases and checks in the code. Lastly, a handful of the school boards posted their case data in the form of PDF documents. We never found an acceptable solution for identifying and parsing out embedded tables in these documents, and so these school boards were monitored by hand.

### Tip 9: Create a clearly advertised channel for grassroots crowdsourcing

JotForm, Google Forms, or other survey tools are very amenable to grassroots data crowdsourcing initiatives. These survey tools allow for efficient and effective data acquisition from remote and external collaborators. Create a strategy to validate the accuracy of the information. CSC provided a form on the project website for contributors to fill basic information about a case and provide a contact e-mail address. Form content was sent to a team email address, which was routinely monitored. A team member would follow up to request supporting documentation, such as PDFs or images showing a letter from a school or public health authority confirming COVID-19 cases or outbreaks. The aforementioned survey tools also allow automatic data transfer into spreadsheets for data management or review by the volunteer team. The link to the submission form should be prominently displayed on all social media profiles as well as on major social media updates. As the forms gain traction for reporting, build in anti-spamming measures into the form such as the requirement of a valid email address to dissuade spammers. To ensure that crowdsourcing is as complete as possible, we recommend periodically comparing regional case counts in the project database to those expected due to local population density. Regions where data collection gaps are anticipated can be monitored by setting e-mail alerts for related news articles, for example, creating Google Alerts for news items with keywords “Nova Scotia + COVID-19 + school”. Confirmed systematic gaps can then be filled by targeted crowdsourcing, such as identifying volunteers from underrepresented geographic regions via social media, or by identifying complementary sources of data.

### Tip 10: Use geocoding libraries and GeoJSON to put data points on a geographical map

Use geocoding libraries to plot data points on a geographical map and visualize location-based data. Geocoding is the process of converting address information, such as street addresses, into geographic coordinates, such as latitude and longitude. Conversion can be performed by geocoding libraries such as *geopy* in Python, open-source options that do not charge fees but can be slow (*photon*), or proprietary options that charge small fees (*mapboxapi*, *ggmap*). In our experience, multiple APIs were necessary to get geolocations for all locations as each API failed for different address formats. Coordinates can then be encoded into GeoJSON format and used to plot data points on a geographical map. GeoJSON is a popular choice for encoding geographic data, as it is easy to encode and decode, and is supported by many mapping frameworks, such as Mapbox, Leaflet, and OpenLayers.

We used provincial databases (e.g., from Ministries of Education) to identify a list of private and publicly funded Canadian schools and, in some instances, obtain geolocations. When geospatial data were missing, we used different APIs to identify geospatial data using street addresses. Despite this, we manually entered geospatial data for approximately 300 schools with invalid geospatial data. The final dataset of Canadian schools with geospatial data can be found in our Zenodo repository (see “Data availability”).

## Outreach

### Tip 11: Find the right social media platform

As discussed elsewhere in this article, a strong social media presence can help grow a team, build collaborations, drive crowdsourcing, and facilitate outreach (Table 2). As demographics vary by platform, evaluate which platforms will advance your cause [16]. A 2022 poll in the US showed that over 90% of journalists use social media in their work, notably Twitter and Facebook, making these platforms a valuable way to get media attention at the time [17]. We recommend that every project identifies the social media platforms currently most used by the target demographic—namely, the audience that will help crowdsource the data, as well as the media—and develop a social media strategy to gain traction on those platforms. On each selected platform, create a profile with a consistent handle and branding, and with a prominent link to your data submission webpage hosted on your project website (see Tip 4). Identify a social media lead in your team, keeping in mind that the tone and content of posts defines your project’s “voice” to your audience. We used R to compile key daily statistics into sets of tweets, complete with emojis using the R *emo* package, into a text file. While tools exist for automated social media posting (e.g., HootSuite, Buffer), we opted to manually post each tweet thread and Facebook post as it was simpler for us. Create a schedule for routine data and social media updates. As online harassment of science communicators is highly prevalent and formal support may be negligible, create a team policy to mute or block trolls and check in on targeted team members [18,19].

### Tip 12: Give broadcast and print media interviews

Engaging with news media can be a powerful message amplifier, which requires development of science communication skills. The CSC project team collectively provided nearly 40 interviews on print and broadcast media. Journalists generally approached team leads via email or social media, requesting interviews around a particular policy development. Decide whether engaging with a media outlet and interviewer will further the cause of the group, and speak to colleagues with previous relevant experience. If you decide to proceed, it is useful and customary to request information about the interview such as duration, name of interviewer, and for a list of potential questions or theme of questions.

Universities usually provide resources and workshops for media training, making related websites a good first stop to help prepare for interviews. Here is our advice to help prepare for a media interview:

- Create a shared cheat sheet containing the interview questions and responses.
- Craft short, memorable sound bites to convey key messages.
- The interviewee represents the team, so use the team’s diverse expertise to ensure that assertions are fact based; add citations in your cheat sheet for reference.
- Responding to questions is as much an opportunity to communicate key advocacy points to the public, as it is about answering the actual question. If none of the questions seem to directly touch on advocacy points, don’t be shy about using your response to communicate your points anyway: you have the platform!
- Anticipate impromptu questions, especially if there are rapid developments, there is controversy, or if the interviewer is known for being spontaneous.
- Share responses with the broader team for feedback.
- Rehearse responses out loud to eliminate awkward phrasing, practicing with someone if needed.
- For syndicated radio with back-to-back interviews, consider splitting interviews with a colleague.
- Promote interviews on social media well in advance to increase your audience and plan to record the interview if it is an option; for example, software such as Audacity can be used to record streaming radio.

On the day of the interview:

- Have team members supporting you, if only virtually, and listening to the live broadcast. The moral support is important if you are still building familiarity with giving interviews, as the experience can be daunting. In our experience, most journalists and radio hosts are skilled interviewers that try to put the interviewee at ease.
- Be aware of the political leanings of the media outlet and anticipate the tone accordingly.
- Avoid inserting personal opinions as you are representing the group, and be frank about admitting when you don’t know the response to a question.
- And, finally, expect spontaneous questions, and be genuine in your response. At this point, you have rehearsed your key points, and one option is to work your way back to the take-home messages.

After the interview, debrief with team members in your group chat, sharing the emotional experience, discussing potential pain points, and formulating a plan for the next interview.

### Tip 13: Build partnerships with industry to improve your platform

**Fig 4:**
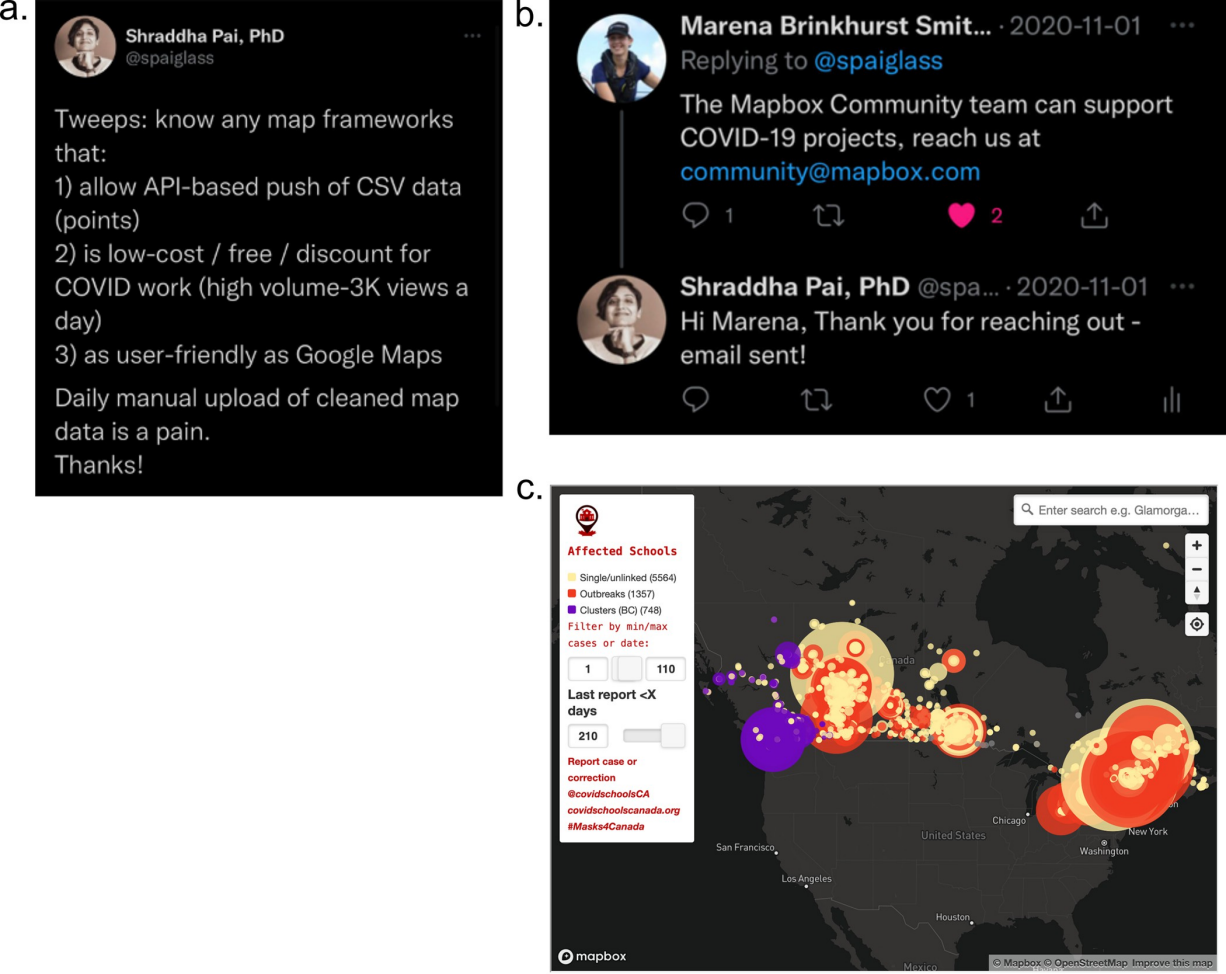
**Left:** The call we put out on social media that led to the connection with Mapbox (**Top Right**). **Bottom right:** Javascript-based map created by partnership with Mapbox. Map generated using MapBox (https://www.mapbox.com/).

While open-source solutions are more powerful than ever, these may not achieve the scalability or ease-of-use required for your project. Consider partnering with a company that offers a free tier option for volunteer projects and not-for-profit projects. For example, the CSC Project initially started entering data points on a custom Google Map, but that solution had limits on data points in a given map layer, and limited automatability. Google for Nonprofits was not an option as this required the group to have a registered non-for-profit tax designation to qualify, which our grassroots volunteer group did not have. To find a scalable solution available to our project, we put out a call on Twitter, which connected us with a Mapbox representative (Fig 4). We learnt that our project was eligible for their free tier of usage, which was ample for our needs. Mapbox provided one-on-one technical support to write the initial code to create an interactive Javascript-based map with the team’s desired features, such as the ability to scale data points by cluster size and limit cases by date range [20]. The updated map was more useful for interaction and data updates could be fully automated.

The project’s relationship with Mapbox is a great example of building ties with like-minded groups to mutual benefit. Since the team was operating without any funding, we approached a few organizations to ask if they would consider supporting the project; while some declined, Mapbox agreed. At that point, our parent volunteer group, Masks4Canada, had already been given a free Pro version of Slack. A different offshoot group of Masks4Canada, Community Access to Ventilation (CAVI, https://www.cavico2.com), decided to incorporate as a not-for-profit entity, and accordingly received discounts for Zoom and Docusign. CAVI also worked with the Canadian distributor of Aranet4 CO2 monitors to secure a discount for public libraries. There are therefore multiple routes to partnerships, based on resources the team decides to invest.

### Tip 14: Know when to end the project and be open to future collaborations

The lifetime of a grassroots volunteer project depends on the ability of the team to sustain the workload, the perceived impact of the work at that point in time, and the availability of other projects to fill a similar need. Team leads need to anticipate a turning point when the effort to sustain a project outweighs its public impact and devise a timeline to wrap up, or sunset, the project. Create a clear timeline for wrap-up with team members, outlining each member’s task list. Freeze changes to data files at a predetermined date and revoke write access. Freeze social media accounts with a final post linking to the project website, where contact information for team leads is prominently displayed, as are links to the software and data made available by the project.

The data your team has crowdsourced is a valuable resource for future study. Create a plan for a final cleaning of the data, documentation, and for its storage for posterity, using resources such as Zenodo, which provide a citable digital object identifier (DOI). Create a framework by which team members will receive credit for future publications where the opportunity arises. Encourage team members to create an Open Researcher and Contributor Identifier (ORCID; orcid.org), a standard in research and scholarship to track contributions to works such as publications and reviews. Create a shareable master document with the names, ORCIDs, current affiliations, and contact information of all team members. Where a collaboration provides the opportunity to get credit for a publication, negotiate the addition of a “team author” entity on the paper, to which ORCIDs of team members are linked.

## Code and data availability

All CSC-related code are publicly available at our GitHub page at: https://github.com/covidschoolscanadaORG. The pipeline for automated data cleaning, analysis, visualization, and tweet generation are available at https://github.com/covidschoolscanadaORG/covidschoolscanada. Web scraper scripts are available at https://github.com/covidschoolscanadaORG/CovidSchoolScraper. Code for the project website is available at https://github.com/covidschoolscanadaORG/covidschoolscanada.github.io

The final freeze of the project data is available at Zenodo (doi: 10.5281/zenodo.7651460).

## Supporting information

S1 Text URLs for resources in Table 2. (DOCX)
