# Supplementary material for: Fourteen quick tips for crowdsourcing geographically linked data for public health advocacy
Source: PLoS Comput Biol. 2023 Sep 21;19(9):e1011285. doi: 10.1371/journal.pcbi.1011285 (PMC10513213; doi:10.1371/journal.pcbi.1011285)
Supplement: S1 Text — (DOCX) [file pcbi.1011285.s001.docx]

S1 Text

# URLs for Resources in Table 2

**Amazon Web Services:** <https://aws.amazon.com/>

**Audacity:** <https://www.audacityteam.org/>

**Boto for Amazon Web Services:** <https://boto3.amazonaws.com/v1/documentation/api/latest/index.html>

**Buffer:** <https://buffer.com/>
**dplyr:** <https://dplyr.tidyverse.org/>

**Dropbox:** <https://www.dropbox.com/>

**Eleventy:** <https://www.11ty.dev/> ; <https://www.11ty.dev/docs/getting-started/>

**Emo R Package:** <https://github.com/hadley/emo>
**Excel:** <https://www.microsoft.com/en-ca/microsoft-365/excel>

**Facebook:** <https://www.facebook.com/>

**geopandas:** <https://geopandas.org/> , <https://github.com/geopandas/geopandas>

**ggmap in R:** <https://towardsdatascience.com/a-guide-to-using-ggmap-in-r-b283efdff2af>

**ggplot in R:** <https://ggplot2.tidyverse.org/>

**GitHub:** <https://github.com/>

**Gmail:** <https://mail.google.com/>

**googleAuthR in R:** <https://cran.r-project.org/web/packages/googleAuthR/vignettes/google-authentication-types.html>

**Google Alerts:** <https://www.google.com/alerts>

**Google Drive:** <https://www.google.com/intl/en_ca/drive/#overview>

**Google Maps:** <https://www.google.com/maps>

**Google Sheets:** <https://www.google.ca/sheets/about/>

**Hootsuite:** <https://www.hootsuite.com/>

**Jekyll:** <https://jekyllrb.com/> ; <https://jekyllrb.com/docs/step-by-step/01-setup/>

**Mapbox:** <https://www.mapbox.com/> , <https://walker-data.com/mapboxapi/>

**Mapbox tutorials:** <https://docs.mapbox.com/help/tutorials/>

**Numpy in Python:** <https://numpy.org/>

**Perl:** <https://www.perl.org/>

**Perl Packages:** <https://www.geeksforgeeks.org/packages-in-perl/>

**Puppeteer:** <https://pptr.dev/>

**Photon:** <https://photon.komoot.io/> , <https://github.com/komoot/photon>

**Python:** <https://www.python.org/>

**R:** <https://www.r-project.org/>

**readxl:** <https://readxl.tidyverse.org/>

**Selenium in Python:** <https://selenium-python.readthedocs.io/>
**Slack:** <https://slack.com/>
**Zoom:** <https://zoom.us/>
**TikTok:** <https://www.tiktok.com/>

**WhatsApp:** <https://www.whatsapp.com/>

**xlsx:** <https://cran.r-project.org/web/packages/xlsx/xlsx.pdf>

**Zenodo:** <https://zenodo.org/>
